# The pharmacogenomics of carbamazepine-induced cutaneous adverse drug reaction in the South of Vietnam

**DOI:** 10.3389/fphar.2023.1217516

**Published:** 2023-07-13

**Authors:** Ai-Hoc Nguyen, Chonlaphat Sukasem, Quy Ngoc Nguyen, Hong Tham Pham

**Affiliations:** ^1^ Department of Pathology, Division of Pharmacogenomics and Personalized Medicine, Faculty of Medicine Ramathibodi Hospital, Mahidol University, Bangkok, Thailand; ^2^ Laboratory for Pharmacogenomics, Somdech Phra Debaratana Medical Center (SDMC), Ramathibodi Hospital, Bangkok, Thailand; ^3^ Department of Pharmacy, Nhan Dan Gia Dinh Hospital, Ho ChiMinh City, Vietnam; ^4^ Pharmacogenomics and Precision Medicine Clinic, Bumrungrad International Hospital, Bangkok, Thailand; ^5^ Bumrungrad Genomic Medicine Institute (BGMI), Bumrungrad International Hospital, Bangkok, Thailand; ^6^ Department of Pharmacy, Nguyen Tat Thanh University, Ho ChiMinh City, Vietnam

**Keywords:** pharmacogenomics, Vietnam, HLA-B*15:02, SJS, MCARs, SCARs, epileptic, carbamazepine

## Abstract

**Background:** The relationship between HLA-B*15:02 and Severe Cutaneous Adverse Reactions was rigorously examined in Japanese, Han Chinese, Thais, and Caucasians. However, the number of studies about this topic in Vietnamese population is still limited and mostly focuses on the North of Vietnam.

**Objective:** This study aims to clarify the genetic culprit of SCARs in Vietnamese population, particularly in the South of Vietnam, and to validate our result by a meta-analysis about this topic in Vietnamese.

**Method:** A retrospective case-control study with 37 patients treated with carbamazepine monotherapy. Statistical calculation and meta-analysis were performed by R software.

**Result:** HLA-B*15:02 increases the risk of SJS 12.5 times higher in CBZ-treated patients (*p*-value = 0.017). However, this allele has no impact on MCARs (Mild Cutaneous Adverse Reactions) of CBZ. The number needed to test and the number needed to genotype is two and nine patients respectively.

**Conclusion:** This study recommends more investigations about the cost-effectiveness of this test to accelerate the protection of Southern Vietnamese from SCARs.

## 1 Introduction

George Snell (Snell, 1981), a Nobel laureate, discovered the Major Histocompatibility Complex (MCH) in 1948. Ten years later, the first Human Leucocyte Antigen (HLA) was detected (Thorsby, 2009). Since then, HLA genes have been gradually unveiled to be one of the most sophisticated genes with over 35,000 alleles confirmed by the time of this study (Barker et al., 2023). Different alleles render different amino acids in MCH molecules, where the antigen presentation happens in a manner of specificity. MCH contains two major classes designated as HLA class I and HLA class II, which are respectively responsible for the endogenous and exogenous pathways. Moreover, the polymorphism of HLA genes plays a crucial role in the development of Steven-Johnson (SJS) and Toxic epidermal necrolysis (TEN).

The diversity of genes encrypted for HLA protein is not only reflected in one single ethnicity, where this diversity allows a wide range of antigens to be recognized and responded to, but also reflected in the differences between ethnicities. Most of the genetic causes of Carbamazepine-induced SCARs were investigated in developed Asian countries, such as Han Chinese (Wang et al., 2011; Zhang et al., 2011), Thais (Tassaneeyakul et al., 2010; Sukasem et al., 2018), and Taiwanese (Chen et al., 2011). However, Southeast Asian populations have received less attention, especially in the South of Vietnam where the flow of immigration created an admixture of crowded population. The South of Vietnam is the home of around 40 million people (2023) with diverse ethnicities such as Kinh Vietnamese, Khmer Krom, Cham Vietnamese, etc. In Caucasians, Japanese, and Koreans, even though a myriad of meticulous research has been conducted about HLA-B*1502, this allele is reported to be rare and not associated with statistically significant risk in these populations. In contrast, the HLA-B*15:02 allele was believed to have the highest allele frequency and fatal risk in the general population of Southeast Asia (Moutaouakkil et al., 2019).

Indeed, the relationship between HLA-*B-15:02 and SCARs was rigorously examined in Japanese, Han Chinese, Thais, and Caucasians. However, the number of studies about this topic in Vietnamese population is still limited and mostly focuses on the North of Vietnam (Van Nguyen et al., 2015; Van Nguyen et al., 2022). Given that Vietnam is a highly populated and racially diverse country, which can create genetic heterogeneity, more research is needed to fully understand the genetic predisposition of Vietnamese, especially those in the south of Vietnam. Unfortunately, as an economically developing area, Vietnam faces tremendous financial barriers in scientific research, which may result in the most vulnerable population possibly receiving the least pre-emptive protection.

HLA-B*15:02 is not only important in the treatment of Carbamazepine, but also in its structural analog, Oxcarbazepine (Phillips et al., 2018). The cross-reactivity of HLA-B*1502 contributes to the improvement in cost-effectiveness, making this allele a worthy investment for personalized therapy.

In this study, we aimed to clarify the genetic culprit of SCARs in the Vietnamese population, particularly in the South of Vietnam. We also compare the associated risk and genetic patterns in the population between the south and the north of Vietnam or other countries.

## 2 Methods

### 2.1 Study design and participants

**FIGURE 1:**
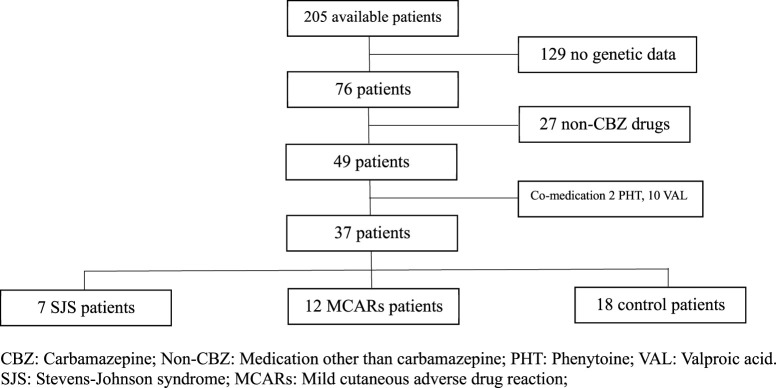
Recruitment process of epileptic patients.

This is a case-control study with the control group defined by patients tolerant with CBZ. The study was conducted in the Department of Neurology at NDGD Hospital from 1 January 2019 to 31 December 2020. The analysis included a total of 7 cases of CBZ-induced SJS, 12 cases of CBZ-induced MCARs, and 18 cases of CBZ-tolerant control as shown in Figure 1.

All patients were recruited retrospectively with the definitions based on the information written in the medical records. The definition of CBZ tolerant is the patients who administered CBZ and the attending physician did not write any diagnosis of SJS or other skin manifestations of allergy. The definition of CBZ-induced SJS is the patients who administered CBZ and the attending physician wrote the diagnosis of SJS. The definition of CBZ-induced MCARs is the patients who administered CBZ and the attending physician wrote the diagnosis of itching skin, hypersensitivity reaction, CBZ allergy, anaphylaxis level 1, skin reaction, or symptomatic allergy. The patients who administered both CBZ and PHT were excluded from this study, only epileptic patients treated with monotherapy CBZ were included. Patients without information of genotype were also excluded from this study. The diagnosis of SJS/TEN was performed by certified dermatologists in accordance with the hospital’s guidelines, which included the following criteria: 1) Onset of symptoms within 2 months of initiating CBZ. 2) Presence of a rash affecting the face, upper body, limbs, or spreading extensively across the entire body. 3) Mucosal damage observed in at least two natural cavities, such as the eyes, nose, mouth, vagina, or anus. 4) Skin detachment (10% for SJS, 30% for TEN) or the presence of Nikolsky’s sign.

### 2.2 Genotyping methods

Within 76 patients with available DNA, 30 DNA samples were sent to the genotyping service at the laboratory of Pharmacogenomic and Personalize Medicine (PPM) of Ramathibodi Hospital Mahidol University, Bangkok, Thailand, 46 DNA samples were sent to the genotyping service at University Medical Center (UMC), University of Medicine and Pharmacy at Ho Chi Minh city. The genotyping method used at PPM was Luminex™ flow cytometry. In brief, the sample DNA binds complementarily to a panel of probes, which was designed with known nucleic sequences. The fluorescence detection technology was used to identify successful binding complexes, and hence, identify the sequence and genotype of the sample. The genotyping method used at UMC was real-time Polymerase Chain Reaction (real-time PCR), using Tagman™ genotyping assay.

### 2.3 Statistical analysis

All statistical analyses were done by R software version 4.2.3 (R Foundation for Statistical Computing, Vienna, Austria). Student’s t-test was used to compare the differences between 2 independent groups. Chi-squared tests were used to compare the ratio of males and females between 2 groups; Fisher’s exact test was employed to compare the risk of alleles between the cases and the controls.

### 2.4 Meta-analysis

**FIGURE 2:**
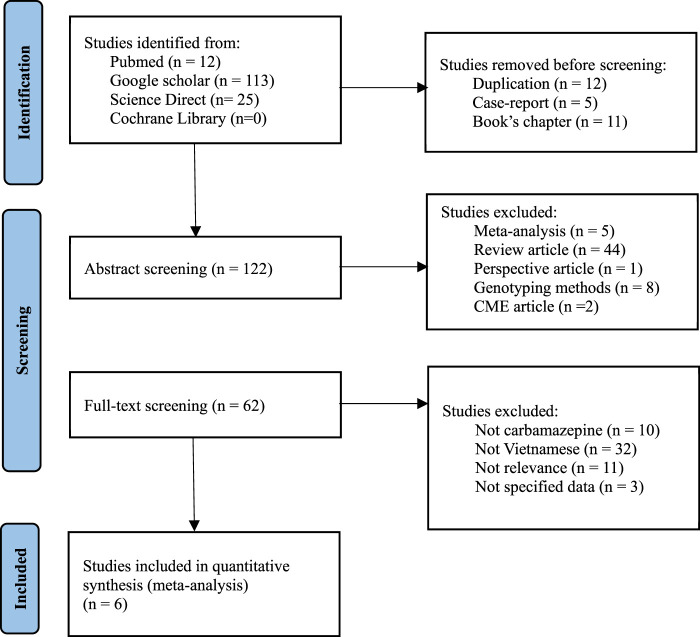
PRISMA flowchart for identification of eligible studies.

This analysis aimed to compare our conclusion with the Vietnamese population in the both the North and the South by evaluating the impact of HLA-B*15:02 in Vietnamese epileptic patients treated with CBZ, utilizing various databases such as Pubmed, Google Scholar, Cochrane Library, and ScienceDirect (Figure 2). The keywords used were “HLA-B*15:02 Vietnamese”, “HLA-B*15:02 Vietnam’’, “SJS”, “Steven-Johnson Syndrome”. To select the suitable studies, the selection criteria were: 1) Containing the case-control analysis of HLA-B*15:02 and SJS, 2) Targeting Vietnamese epileptic patients, 3) Including the clear definitions of case and control with corresponding numbers, and the exclusion criteria were: 1) Duplicating studies, 2) Researching about the development of genotyping methods in Vietnam, 3) Being a case report or cross-sectional study. Data analysis and visualization were performed on Rstudio, using random effect with a *p*-value lower than 0.05 is considered statistically significant, and with I^2^ higher than 50% was defined as heterogeneous. The estimation method used for the random effect is restricted maximum likelihood.

### 2.5 Ethics approval

This study was approved by the Ethics Committee of NDGD Hospital, Ho Chi Minh City, Vietnam, under approval number 23-2015/CN-HĐĐĐ, on 13 October 2015. As we used only retrospective data from health records while also maintaining patient confidentiality, no informed consent was required for this study.

## 3 Result

### 3.1 Genetic and demographic information of patients

Table 1 describes the characteristic of the participants. The age of patients was widely distributed from 25 to 85 years old. The separation of gender was fairly equal between males (21 patients) and females (16 patients) in total participants. However, there was a higher proportion of males in the SJS group. The percentage of males respectively was 50% and 55.6% in the group of CBZ-induced MCARs and CBZ-tolerant control. All three groups contained patients with chronic diseases, such as hypertension or dyslipidemia. Nearly 30% of epileptic patients had a medical history of cerebral infarction or brain hemorrhage.

**TABLE 1:** Demographic data of recruited epileptic patients.

	CBZ-induced SJS (n = 7)	CBZ-induced MCARs (n = 12)	CBZ-tolerant control (n = 18)	*p* values ^Ŧ^
Gender (number; percent)				
Male	5 (71%)	6 (50%)	10 (55.6%)	0.785; 1.000
Female	2 (29%)	6 (50%)	8 (44.4%)	
Age (years)				
Min	30	25	31	0.4813; 0.3227
Average	51	49.8	56.2	
Max	83	85	81	
Group of age				
25–45	2	6	6	
46–65	4	3	9	
66–87	1	3	3	
Height (cm)				
Min	150	148	150	0.5821; 0.3097
Average	164.6	159.6	162.6	
Max	173	172	176	
Weight (kg)				
Min	51	43	47	0.5954; 0.4199
Average	62.3	57	59.7	
Max	86	73	80	
Diagnosis other than epilepsy				
Hypertension	1	1	7	
Dyslipidemia	1	2	6	
Implication of cerebral infarction	1	2	5	
Implication of brain hemorrhage	2	0	1	
Cerebral vascular insufficiency	0	0	5	
Liver disease	2	1	0	
Osteoporosis	1	0	1	

SJS: Stevens-Johnson syndrome; Ŧ: The first *p*-value is between CBZ-induced SJS, and tolerant control. The second *p*-value is between CBZ-induced MCARs, and tolerant control. *p* values were received from the Student’s t-test (except for gender variables, which used the Chi-squared test). A *p*-value lower than 0.05 are considered statistically significant (two-sided); cm: centimeter; kg: kilogram.

Among twelve carbamazepine-induced MCARs patients, five patients were diagnosed with itching skin and hypersensitivity reactions, six patients were diagnosed with symptomatic allergy and skin reactions of allergy, and one patient was diagnosed with anaphylaxis level 1 due to CBZ allergy. It is important to note that none of the twelve patients exhibited symptoms specific enough to be classified as SJS/TEN.

### 3.2 The frequencies of *HLA-B* alleles detected in Southern Vietnamese

Table 2 shows the HLA allele polymorphism in the epileptic southern population of Vietnam, where a total of 22 alleles were observed. The most popular *HLA-B* alleles are *15:02* (20%), *38:02* (10%), and *46:01* (10%). There is a 5% of frequency in each of the following alleles: 7:05; 18:01, 37:01, 57:01. The rest 15 alleles account for around 1.7%–6.7% of the whole population.

**TABLE 2:** The frequencies of detected *HLA-B* alleles in the population of Southern Vietnamese.

Allele	Freq	Allele	Freq	Allele	Freq	Allele	Freq
7:05	0.05	15:25	0.033	38:02	0.1	51:01	0.017
13:01	0.017	18:01	0.05	39:01	0.017	53:04	0.017
15:01	0.017	27:04	0.033	40:01	0.067	57:01	0.05
15:02	0.2	27:06	0.033	44:03	0.017	58:01	0.033
15:08	0.017	35:05	0.033	46:01	0.1		
15:12	0.033	37:01	0.05	50:01	0.017		

*HLA-*B: Human leucocyte antigen class 1 B; Freq: Frequency.

### 3.3 The increased risk patients carrying HLA-B*15:02 in Southern Vietnamese

Table 3 illustrates that in the Southern Vietnamese population, the HLA-B*15:02 allele is a statistically significant risk factor for SJS (*p* = 0.017), but not for MCARs (*p* = 0.660). The odd ratio of the HLA-B*15:02 allele is 12.5. In other words, the carriers of this allele have 12.5 times higher risk than non-carriers, in terms of SJS. The sensitivity and specificity are 71.4% and 83.3% respectively, meaning that the hospital’s protocol can correctly identify 71.4% of SJS patients, and correctly identify 83.3% of non-SJS patients. Besides, the PPV and NPV are 62.5% and 88.2% respectively. These two parameters are interpreted in the context that the results of HLA-B*15:02 are available. In these cases, if the result of a patient is positive, the possibility for this patient to have SJS is 62.5%, and if the result of a patient is negative, the possibility for this patient to not have SJS is 88.3%. Moreover, the NNT and NNG are respectively 2 and 9, which confirms that to protect 1 patient from SJS, the intervention of replacing CBZ with an alternative drug has to be done in 2 patients, or the genetic test has to be done in 9 patients.

**TABLE 3:** The association and odd ratio of *HLA-B* alleles.

*HLA-B*	Case	Tolerant control	OR (95% CI)	*p* values	Sens (%)	Spec (%)	PPV (%)	NPV (%)	NNT	NNG
**SJS**										
15:02	5/2	3/15	12.50 (1.60, 97.65)	0.017	71.4	83.3	62.5	88.2	2	9
**MCARs**										
15:02	3/9	3/15	1.67 (0.28, 10.09)	0.660	25	83.3	50	62.5	8	40

SJS: Stevens-Johnson syndrome; MCARs: Mild Cutaneous Adverse Reactions; OR: odd ratios; 95% CI: 95% confident interval; *p* values were calculated by Fisher exact test with the significance threshold of 0.05; Sens: Sensitivity; Spec: Specificity; PPV: positive predictive value; NPV: negative predictive value; NNT: number needed to treat; NNG: number needed to genotype.

Out of the twelve cases of allopurinol-induced MCARs, three patients tested positive for HLA-B*15:02. While the strength of evidence is not large enough to be statistically significant, it is worth noting that the rate of HLA-B*15:02 in the MCARs group is higher compared to the group of tolerant controls.

### 3.4 Meta-analysis

**FIGURE 3:**
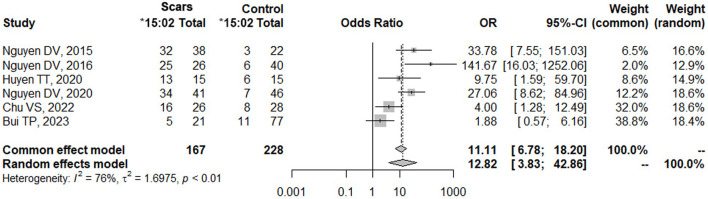
Forest plot of the association between HLA-B*15:02 and SCARs in Vietnamese.

A meta-analysis was performed about the risk of HLA-B*15:02 and SCARs in Vietnamese with a total of 6 studies (Van Nguyen et al., 2015; Van Nguyen et al., 2017; Huyen et al., 2020; van Nguyen et al., 2021; Chu, 2022; Bui et al., 2023). All these 6 studies were conducted and recruited patients exclusively in the northern region of Vietnam, specifically at Bach Mai Hospital, Tam Anh Hospital (Ha Noi branch), and the National Hospital of Dermatology and Venerology. Surprisingly, no eligible studies conducted in the southern region of Vietnam were identified or included in this meta-analysis (Supplementary Table S1). Interestingly, a recent study by Bui TP et al. (Bui et al., 2023) reported a negative association between HLA-*B15:02 and SCARs in Vietnamese in January 2023. However, our analysis using pooled data from all six studies showed a strong positive association between HLA-B*15:02 and SCARs, as determined by both common effect and random effects models (Figure 3). Carriers of HLA-B*15:02 had 12.82 times higher odds of developing SCARs compared to non-carriers (*p*-value <0.01). The odd ratio calculated from previous studies in the meta-analysis was consistent with the odd ratio in the current study.

## 4 Discussion

This is the first case-control study to investigate the pharmacogenomics of CBZ-induced SJS in Southern Vietnam. Based on the evidence that the pharmacogenomic test can protect one case of SJS with every two interventions or every nine genetic tests, we recommend implementing this test in the population of Southern Vietnamese.

In the population of Southern Vietnamese, our study reported a higher frequency of the HLA-B*15:02 allele, 20% compared to 11.88% in a study of the general population of Southern Vietnamese by Do MD et al. (Do et al., 2020). In the population of Northern Vietnamese, the frequency of HLA-B*15:02 in epileptic patients varies between studies 13.5% (Van Nguyen et al., 2015), 15.2% (van Nguyen et al., 2021), 17.7% (Bui et al., 2023), and 41.7% (Huyen et al., 2020). The possible explanation may be due to the different regions or the small sample size, which can only reflect a part of the whole population. Interestingly enough, Que TN et al. (Que et al., 2022) reported a frequency of 15.11% of HLA-B*15:02 with a sample size of 3750 participants, who were recruited in Northern and North-central Vietnam (Que et al., 2022). Besides, we found the frequencies of HLA-B*38:02 and *46:01 to be equally 10%. Comparatively, Do MD et al. (Do et al., 2020) reported these two alleles to be 7.92% and 9.41% respectively. Que TN et al. (Que et al., 2022) also reported these two alleles to be 7.29% and 10.7% respectively.

Regarding global populations, Southeast Asians are regarded as having the highest frequency of HLA-B*15:02 in the world (Moutaouakkil et al., 2019; Van Nguyen et al., 2019). Indeed, numerous studies published the frequency of HLA-B*15:02 in their own country, such as 15.11% in Thailand (Zhang et al., 2011), 32.8% in Indonesia (Khosama, 2017), 8.3% in Malaysia (Chang et al., 2011), and 5.7% in Singapore (Middleton et al., 2004). Moving toward northeast Asia, this frequency gradually decreases, such as 7.3% in Southern Chinese (Trachtenberg et al., 2007), 1.9% in Northern Chinese (Hong et al., 2005), 7.7% in Taiwanese (Chen et al., 2011), 10.2% in Hongkong (Middleton et al., 2004), 1.7% in Japanese (Ikeda et al., 2010) and 0.4% in South Koreans (Kim et al., 2011). In contrast, HLA-B*15:02 is seemingly absent in Caucasians, such as French (Gourraud et al., 2015), and the United States (Maiers et al., 2007).

The clinical implication of HLA-B-*15:02 is specifically related to SJS and not MCARs, even though both are the cutaneous manifestations of allergy. This observation can be explained by recent immunologic findings that the CBZ hyperactivity reaction is not solely dependent on the specificity of HLA, but also the specificity of the T-cells and their receptors (Ko et al., 2011; Ko and Chen, 2012). This evidence also explains why three patients in our study tested positive for HLA-B*15:02 but did not develop SJS.

Pharmacogenomics and personalized medicine are well-known to be ethnicity-specific. Even though in the same country, the genetic diversity could be significant and should not be ignored. This is especially true in the case of Vietnam, with a population of 100 million or 54 ethnicities, concentrated mainly in two metropolitan and healthcare centers of the north and the south. The absence of medical evidence in an ethnic group can impede the clinical implementation of a beneficial intervention. This is not only a medical issue, but also an ethical problem to have an equitable healthcare system (Patrinos et al., 2023). Besides the genetic differences, the socioeconomic and academic differences between regions also play an important role in pharmacogenomic research and implementation to ensure cost-effectiveness in resource-limited hospitals (Nagar et al., 2019).

Several limitations need to be rectified by future research. Firstly, the patients were genotyped by two genotyping methods, which may have increased the potential for bias. The decision to employ these methods was necessitated by the challenging circumstances presented by the COVID-19 pandemic. As a result, the HLA-B*15:02 genotyping had to be conducted at a different hospital. Therefore, the better choice would be to use only one genotyping method. Secondly, the sample size is relatively small, larger studies should be conducted to clarify the finding in this study more thoroughly, especially for the outcome of MCARs in Southern Vietnamese. Thirdly, the exclusion of patients without genotyping results is another limitation, and future endeavors with more financial support should aim to include more patients to obtain a more comprehensive understanding of the real-world population.

In conclusion, this study confirms the association between CBZ-induced SJS and HLA-B*15:02 in Vietnamese, particularly in the Southern population. Therefore, it is recommended to perform further studies about the cost-effectiveness of this test to accelerate the protection of Southern Vietnamese from SCARs.

## Data Availability

The raw data supporting the conclusions of this article will be made available by the authors, without undue reservation.
